# Effect of Extraction Method on the Bioactive Composition, Antimicrobial Activity and Phytotoxicity of Pomegranate By-Products

**DOI:** 10.3390/foods11070992

**Published:** 2022-03-29

**Authors:** Lara Campos, Luana Seixas, Susana Dias, António M. Peres, Ana C. A. Veloso, Marta Henriques

**Affiliations:** 1 Polytechnic Institute of Coimbra, Coimbra Agriculture School, Bencanta, 3045-601 Coimbra, Portugal; sudias@esac.pt (S.D.); mhenriques@esac.pt (M.H.); 2 CERNAS, Research Centre for Natural Resources, Environment and Society, Coimbra Agriculture School, Bencanta, 3045-601 Coimbra, Portugal; 3 Polytechnic Institute of Coimbra, ISEC, DEQB, Rua Pedro Nunes—Quinta da Nora, 3030-199 Coimbra, Portugal; luana.seixas00@gmail.com (L.S.); anaveloso@isec.pt (A.C.A.V.); 4 Centro de Investigação de Montanha (CIMO), ESA, Instituto Politécnico de Bragança, Campus de Santa Apolónia, 5300-253 Bragança, Portugal; peres@ipb.pt; 5 CEB—Centre of Biological Engineering, University of Minho, Campus de Gualtar, 4715-057 Braga, Portugal; 6 LABBELS—Associate Laboratory, Braga/Guimarães, Portugal

**Keywords:** sonication-assisted extraction, solvent extraction, *Punica granatum* L., pomegranate peels, pomegranate seeds, antimicrobial activity, phytotoxicity

## Abstract

Pomegranate by-products can be an asset to the food industry due to the richness in bioactive and antimicrobial compounds. This work studied the influence of conventional solvent and sonication-assisted extraction methods on the bioactive profile, antimicrobial properties, and phytotoxicity effect of the peels and seeds extracts from Acco, Big Full, and Wonderful pomegranate cultivars. The bioactive composition of the extracts was evaluated for the content of total phenolics, total flavonoids, and antioxidant activity (expressed as the half-maximal inhibitory concentration—IC_50_) by spectrophotometric methods, while the tannins were determined by titration and the anthocyanins were estimated by the pH-differential method. For the evaluation of the antimicrobial activity, the disk diffusion method of Kirby-Bauer was adapted through inhibition halos against *Escherichia coli*, *Pseudomonas aeruginosa*, *Staphylococcus aureus*, *Bacillus cereus*, and *Yarrowia lipolytica*. The extracts’ phytotoxicity was evaluated in vitro on garden-cress seeds. Extracts from conventional extraction were richer in total phenolics, expressed as gallic acid equivalents (0.16–0.73 mg GAE/mg extract), while those from sonication-assisted extraction had higher contents of total flavonoids, expressed as catechin equivalents (0.019–0.068 mg CATE/mg extract); anthocyanins, expressed as cyanidin-3-glucoside (0.06–0.60 µg C3G/mg, dry basis); and antioxidant activity (IC_50_, 0.01–0.20 mg/mL). All extracts were more effective against Gram-positive bacteria and yeasts than Gram-negative bacteria. In general, the sonication-assisted extracts led to higher inhibition halos (8.7 to 11.4 mm). All extracts presented phytotoxicity against garden-cress seeds in the tested concentrations. Only the lowest concentration (0.003 mg/mL) enabled the germination of seeds and root growth, and the sonication-assisted extracts showed the highest Munoo-Liisa vitality index (51.3%). Overall, sonication-assisted extraction obtained extracts with greater bioactive and antimicrobial potential and less phytotoxicity.

## 1. Introduction

In the European Union, food waste generated during food processing represents up to 39% of the total food waste [1]. A large amount of the food waste corresponds to by-products that are rich in valuable compounds. Pomegranate (*Punica granatum* L.) is a fruit grown all over the world, predominantly in western Asia and the Mediterranean region [2]. The proportions of peel:arils:seeds of the pomegranate fruit are, respectively, 50:40:10 [3,4], which means that during pomegranate processing, about 60% of the fruit is produced as a by-product that could potentially be discarded [5], representing additional costs for its disposal [6]. However, these by-products contain important amounts of phenolic compounds (such as flavonoids and tannins), sugars, organic acids, and minerals, and have antioxidant, antifungal, and antibacterial activities [2,3,4,5,7].

Phenolic compounds from plant matrices are responsible for several benefits to human health. These benefits come from the ability of these phytochemicals to alter enzymatic and chemical reactions [8]. Due to the increasing awareness of consumers about the benefits of enhancing their quality of life through the consumption of natural compounds (e.g., prebiotics, probiotics, supplements, dietary fibers, or functional foods), intense research has been carried out on their importance, mechanisms of action, and recovery processes. The recognized biological potential of pomegranate by-products makes them good candidates for reintegration into the industry chain (transformed or incorporated into other products) after the recovery of the compounds of interest [9]. Although there are several methods capable of recovering these compounds, such as supercritical fluid extraction, microwave-assisted extraction, extraction with pressurized liquids, and extraction with pressurized hot water [10], the most applied is conventional solvent extraction.

Conventional extraction methods include organic solvent extraction and distillation [11], with Soxhlet extraction, maceration, and hydrodistillation [12,13] being the classical techniques. These methods generally depend on the effect of solvent, temperature, and extraction time on the matrix. The increase in the extraction temperature promotes the mass transfer and diffusion of the compounds present in the matrix to the solvent and enhances the solubility of the extracted compounds [14].

However, conventional methods often have practical, economic, and social concerns that are difficult to overcome or fail to achieve sustainability. Some of its drawbacks are related to the matrix overheating, which can lead to loss of functionality or stability of the final product (degradation of compounds of interest during extraction) [14,15], high energy consumption (and resources in general) [10], emission of volatile organic compounds and the related polluting effect [15,16], and difficulties in complying with increasingly strict safety regulations. Thus, greener technological processes have emerged, which seem to overcome some of these problems [10,15]. Sonication-assisted extractions, which are a type of ultrasound extraction, can be used for this purpose as the extraction can be completed in less time with high repeatability [15,17].

Ultrasound has been recognized as a potential method of extracting oils, proteins, and bioactive compounds from plants [18] because the propagation of the pressure waves and the resulting cavitation forces disrupts the cell walls and improves the release of substances into the solvent [12,15,19]. This extraction method has the main advantages of not being destructive of active ingredients in plant matrices and intensifying the extraction of bioactive compounds (such as phenolics) [10].

This work aims to compare the efficiency of two extraction methods (conventional and sonication-assisted extraction) on the bioactive quality of the recovered extracts. For this, the phytochemical and antimicrobial potential of the extracts of the peels and seeds of three pomegranate cultivars (Acco, Big Full, and Wonderful) grown in the Alentejo region (southeast Portugal) were assessed through the evaluation of the content of total phenolic compounds (TPC), total flavonoids (TF), tannins (TAN), anthocyanins (ANT), antioxidant activity (AA), inhibition halos against various microorganisms, and, finally, its phytotoxicity towards garden-cress seeds.

On the other hand, to date, few research studies have provided information on the bioactive and antimicrobial potential of pomegranate peels and seeds, namely for fruits grown under Portuguese agroclimatic conditions [20], and no studies have been found for Big Full cultivar worldwide. In this context, it was also intended to contribute with new knowledge in order to strengthen the hypothesis that these by-products can be valorized and used as raw materials for food, pharmaceutical, or cosmetic industries, and, even, for agricultural purposes.

## 2. Materials and Methods

Peels and seeds from Acco, Big Full, and Wonderful cultivars were submitted to two extraction methods: conventional and sonication-assisted extraction, using an ethanol:water mixture (50%, *v*/*v*). The obtained extracts were then analyzed to quantify the TPC, TF, TAN, ANT, and AA. The antimicrobial potential was evaluated through inhibition halos against two Gram-positive bacteria (*Bacillus cereus* and *Staphylococcus aureus*), two Gram-negative bacteria (*Escherichia coli* and *Pseudomonas aeruginosa*), and one yeast (*Yarrowia lipolytica*). The phytotoxicity of the extracts that presented the best bioactive and antimicrobial potential was evaluated against garden-cress seeds.

### 2.1. Pomegranate Cultivars, Peels, and Seeds Recovery and Preparation

Pomegranate fruits from Acco, Big Full, and Wonderful cultivars were supplied by POM Portugal Lda and harvested in the Alentejo region, in the southeast of Portugal (GPS coordinates 37.81717, −8.19534). All pomegranate cultivars were grown under the same climatic conditions and underwent the same agronomic practices (fertilization, irrigation, harvesting, storage, and post-harvest treatments). Although Acco (Akko) and Wonderful are already known and studied cultivars, Big Full is a new and improved pomegranate cultivar (from Acco) and, thus, is less studied.

Several factors can influence the amount of bioactive compounds in a matrix, and the fruit maturation stage is one of them. It was observed that the antioxidant activity decreases as the fruit grows, since the development of the fruit leads to a decrease in the phenolic acids content [21]. In addition, fruit development changes the composition of flavonoids [22]. To limit this possible influence, in this work, all fruits were harvested fully mature, by evaluating the soluble solids content (°Brix) of the juice, which was 15° Brix for Acco, 16° Brix for Big Full, and 15° Brix for Wonderful.

After harvesting, the ripe fruits were transported in appropriate boxes to the Agriculture School pilot plant (Coimbra). Fruits were washed and sorted to remove the rotten ones. Proper fruits were cut in 4 pieces, and the arils were separated from the peels using a grape stem removing machine (COSVAL, Mizar 60, Cosvalinox, Oliveirinha, Portugal). The arils were crushed and squeezed using a pressing machine (Aguinox, Marmorier 30 × 40, Águeda, Portugal), and the pomegranate juice was discarded. The peels and seeds were then coarsely ground and dried in a forced hot air dryer (Conterm Drying Oven 2000210, J.P. Selecta, Spain) at 70 °C for 24 h. The final moisture contents of the peels and seeds were, respectively, 17.8% and 21.2% for Acco; 10.1% and 14.1% for Big Full; and 19.1% and 15.7% for Wonderful. Before extraction, the dry material was finely ground into a powder using a mix grinder (Classic 123, 700 W, Moulinex, Écully, France), packed in sealed plastic bags, and stored at room temperature (20–25 °C) protected from the light. 

### 2.2. Chemicals

Methanol (Ceamed, Lda., Funchal), Folin and Ciocalteu’s phenol reagent (Biochem Chemopharma, Cosne-Cours-sur-Loire, France), sodium hydroxide p.a. (Eka, Netherlands), sodium acetate (Honeywell, Charlotte, CA, USA), sodium nitrite p.a. (Merck, Darmstadt, Germany), indigo carmine (labkem, Spain), potassium permanganate (Acofarma, Madrid, Spain), sodium carbonate anhydrous p.a., potassium chloride (Panreac, Spain), absolute ethanol and sulfuric acid 95–97% (Chem-Lab, Zedelgem, Belgium), gallic acid (GA), catechin (CAT), aluminum chloride (99%), and 2,2-diphenyl-1-picrylhydrazyl (DPPH), purchased from Sigma-Aldrich (St. Louis, MO, USA), were used as chemicals for the extractions and bioactive characterization of the extracts.

Agar Powder (VWR, Lutterworth, UK); amphotericin B solution (Sigma, USA); blank, kanamycin (K) 30UG, and penicillin G (P) 2IU discs (LIOFILCHEM, Roseto degli Abruzzi, Italy); D(+)-glucose monohydrate and yeast extract (Scharlau, Barcelona, Spain); dimethyl sulfoxide (DMSO) and Mueller-Hinton Agar (Merck, Germany); Nutrient Agar (Biolab, Budapest, Hungary); and Nutrient Broth and Peptone (Cultimed, Spain) were used as chemicals for antimicrobial characterization of the extracts.

### 2.3. Pomegranate Peels and Seeds Extraction Methods

Ethanol:water mixture (EtOH 50%, *v*/*v*) was prepared and used as solvent for the extraction of the bioactive compounds from pomegranate peels and seeds powders, and a solid:solvent ratio of 0.02 g/mL was applied. The conventional solid:liquid extraction was conducted for 4 h, in sealed glass flasks under continuous stirring (200 rpm) andimmersed in a water bath at 50 °C. For sonication-assisted extraction, the Q700 sonicator (QSonica, Newtown, CT, USA) equipped with a probe (CL-334, Qsonica, USA) was used and the pomegranate peels and seeds were sonicated at room temperature, for 20 min under a frequency of 20 kHz, which was selected based on the literature data regarding different plant matrices, aiming to avoid the formation of free radicals that is promoted by frequencies greater than 20 kHz [10,23,24,25,26,27]. After extraction, the solid:liquid mixtures were filtered under vacuum. The obtained liquid extracts were used for tannin and anthocyanin content determination. Then, the solvent was evaporated at 90 °C, until ca. 20 mL, using a rotary evaporator (Rotavapor R-210, Buchi, Switzerland) under vacuum. The concentrated extracts were frozen at −18 °C overnight, freeze-dried (UNICRYO MC 4l −60 °C, Uniequip, Planegg, Germany) and stored at −18 °C until further analysis. Four independent extractions were made for each cultivar, type of by-product, and extraction. The extraction yields (EY) were calculated as the mass of the extract recovered from the mass of the dry pomegranate material used for the extraction (mg of extract per 100 mg of dry by-product) and expressed as percentage.

### 2.4. Bioactive Compounds Quantification

#### 2.4.1. Total Phenolic Compounds Determination

The TPC in the pomegranate by-product extracts was determined spectrophotometrically following the method proposed by Singleton and Rossi [28]. The peels and seeds extracts were dissolved in methanol:water (70:30, *v*/*v*) at a concentration of 0.2 mg/mL. Gallic acid was used as standard for the calibration curve, within a concentration range of 0.07–0.70 mg/mL (Abs_750nm_ = 9.729 × TPC − 0.019; *R*^2^ = 0.995). A 200 µL of Folin-Ciocalteu reagent was added to 200 µL of the dissolved extract and placed in a water bath at 40 °C. After 4 min, 1600 µL of 5% Na_2_CO_3_ (*w*/*v*) were added. After 20 min of adding the Folin–Ciocalteu reagent, the extracts were removed from the water bath, and the absorbance was measured at 750 nm on a UV/VIS Spectrometer T80+ (PG Instruments Ltd., Lutterworth, UK). A methanol:water solution (70:30, *v*/*v*) was used as a blank sample. TPC were expressed as mg of gallic acid equivalent per mg of extract (mg GAE/mg extract).

#### 2.4.2. Total Flavonoids Determination

The TF were assessed spectrophotometrically according to the method proposed by Kim et al. [29]. The extracts were dissolved in a methanol:water solution (50:50, *v*/*v*) at a concentration of 0.6 mg/mL. Catechin was used as standard for the calibration curve, within a concentration range of 0.08–0.40 mg/mL (Abs_510nm_ = 2.985 × TF + 0.049; *R*^2^ = 0.990). First, 4 mL of distilled water were added to 1 mL of the extract solution, followed by 0.3 mL of 5% NaNO_2_ (*w*/*v*). After 5 min, 0.3 mL of 10% AlCl_3_ (*w*/*v*) were added to the mixture. Then, 6 min later, 2 mL of NaOH (1 M) and 2.4 mL of distilled water were also added, and the absorbance of the final mixture was measured at 510 nm in a UV/VIS Spectrometer (T80+, PG Instruments Ltd.). A methanol:water solution (50:50, *v*/*v*) was used as blank sample. TF were expressed as mg of catechin equivalent per mg of extract (mg CATE/mg extract).

#### 2.4.3. Tannins Determination

The TAN content was analyzed according to the method described by Atassanova and Christova-Bagdassarian [30], with some modifications. After extraction and vacuum filtration of the solid part, 5 mL of each extract solution were mixed with 5 mL of indigo solution and 150 mL of distilled deionized water. An aqueous solution of KmnO_4_ (0.1 N) was used for titration, until the blue color turns to a golden yellow. A blank test was performed using 5 mL of the extraction solvent EtOH 50%, instead of an extract solution. The tannin content was determined using Equation (1) and expressed as percentage of tannin mass per total sample mass on a dry basis (%, *w*/*w*, db).

(1) $$\%\mathrm{TAN}=\frac{\left(V-V_{0}\right)\times 0.004157\times 100}{W}$$

where, *V* is the volume of KmnO_4_ solution spent for titration of the sample, *V*_0_ is the volume of KmnO_4_ solution spent for titration of the blank, 0.004157 is tannin equivalent in 1 mL of 0.1 N of KmnO_4_ solution, and *W* is the weight of the peels or seeds powder used.

#### 2.4.4. Anthocyanins Determination

Anthocyanin content (ANT) was estimated by the pH-differential method of Sellappan et al. [31] with modifications, using two buffer systems: potassium chloride buffer, pH 1.0 (0.025 M), and sodium acetate buffer, pH 4.5 (0.4 M). An amount of 0.4 mL of each extract solution was mixed separately with 1.6 mL of each buffer, and the absorbance was read at 510 and 700 nm in a UV/VIS Spectrometer (T80+, PG Instruments Ltd.). Distilled water was used as the blank solution to calibrate the spectrometer.

Monomeric anthocyanin pigment concentration in the extract solution was calculated by Equation (2) and expressed as μg of cyanidin-3-glucoside (C3G) per mg of sample (μg C3G/mg sample, db).

(2) $$\mathrm{ANT}\;\left(\mu g\;C3G/\mathrm{mg}\;\mathrm{sample}\right)=\frac{Abs\times MW\times DF\times 1000}{\in \times 1}$$

where, $Abs={\left(Abs_{510\mathrm{nm}}-Abs_{700\mathrm{nm}}\right)}_{pH1.0}-{\left(Abs_{510\mathrm{nm}}-Abs_{700\mathrm{nm}}\right)}_{pH4.5}$, *MW* is the molecular weight of C3G (449.2 g/mol), *DF* is the dilution factor; and ∈ is the molar absorptivity of C3G (26,900).

#### 2.4.5. Antioxidant Activity (DPPH Radical Scavenging Assay)

The AA of the extracts was measured spectrophotometrically [32,33,34]. DPPH was dissolved in EtOH to obtain a concentration of 0.3 mM. The extracts were dissolved, at least in 3 different concentrations, in EtOH 50%. An amount of 2.5 mL of EtOH 50% was used as control and 2.5 mL of the extract solutions were used as samples. Then, 1 mL of DPPH solution was added to the control and samples and left for 30 min protected from light. After this period, the absorbance was measured at 517 nm (UV/VIS Spectrometer T80+, PG Instruments Ltd.). For each sample, a blank with 2.5 mL of the extract solution and 1 mL of EtOH was used. The AA was calculated using Equation (3).

(3) $$\%\mathrm{AA}=\left(1-\frac{Abs_{s}-Abs_{b}}{Abs_{c}}\right)\times 100$$

where, *Abs_s_* is the absorbance of the sample, *Abs_b_* is the absorbance of the blank, and *Abs_c_* is the absorbance of the control.

The IC_50_ (half-maximal inhibitory concentration, i.e., the amount of antioxidant required to decrease the initial DPPH concentration by 50%), was determined by the linear fitting of the data of %AA vs. extract concentrations (%AA must give results below and above of 50%). Finally, the AA of each extract was expressed in terms of IC_50_, in mg/mL.

All the analyses of TPC, TF, TAN, ANT, and AA were performed in duplicates of two independent assays.

### 2.5. Antimicrobial Analysis

#### 2.5.1. Microorganisms’ Activation and Preparation

The antimicrobial evaluation of the extracts was performed against pathogenic Gram-positive bacteria (*Bacillus cereus* ATCC 10876 and *Staphylococcus aureus* ATCC 29213), Gram-negative bacteria (*Escherichia coli* ATCC 25922 and *Pseudomonas aeruginosa* ATCC 27853), and a yeast (*Yarrowia lipolytica* ISA 1774).

Based on previous studies [35,36,37], 10% DMSO (dimethylsulfoxide) was selected to be used as a solvent to study the antimicrobial activity of pomegranate extracts that were diluted to a concentration of 0.30 g/mL.

Before each test, all microorganisms were activated in their respective media and incubated at specific temperature and time. Pathogenic bacteria were incubated at 37 °C for 24 h in Mueller-Hinton Agar (MHA) and yeast at 25 °C for 72 h in GYPA medium (20 g glucose, 10 g yeast extract, 20 g peptone, and 15 g agar powder, for 1000 mL of distilled water). After incubation, the microorganisms were suspended in Nutrient Broth (NB) or GYP medium (for bacteria and yeasts, respectively), in sterile test tubes, measuring their density with a Densichek Plus densitometer (BIOMERIEUX, Linda-a-Velha, Portugal), until reaching 0.5 McFarland (1.5 × 10^8^ CFU/mL for bacteria; 2.0 × 10^6^ CFU/mL for yeast).

#### 2.5.2. Inhibition Halos

To determine the inhibition halos of the extracts against each microorganism was used the disk diffusion method of Kirby-Bauer with adaptations. An amount of 250 µL of the inoculum was placed in a Petri dish with MHA (for bacteria) or GYPA (for yeast). Blank discs (in triplicate) were submerged with 20 µL of each re-suspended extract and placed on top of the inoculum. One blank disc with 20 µL of distilled water was used as a negative control. Kanamycin and penicillin discs were used as positive controls for bacteria, and 20 µL of amphotericin B solution was placed in a blank disc to serve as a positive control for yeast. The Petri dishes were incubated at 37 °C for 24 h for bacteria and at 25 °C for 48 h for yeast. The diameters of the inhibition halos of the extracts and respective controls were measured in millimeters. The effectiveness of the extracts is determined by comparing their inhibition halos with those of the positive control. The results of the inhibition halos allowed to determine the Antimicrobial Activity Index (AAI), according to Equation (4), adapted from Vancheva et al. [38].

(4) $$\%\;\mathrm{AAI}=-1\times \frac{A-E}{A+E}\times 100\;$$

where, *A* is the mean value of the inhibition halo (mm) promoted by the positive control (antibiotic/antifungal) and *E* is the mean value of the inhibition halo (mm) promoted by the extract.

AAI ranges between [−100% to 100%]. AAI equal to −100% means the extract showed no inhibition halos; AAI values in the range ]−100 to 0%[ mean the extract showed smaller inhibition halos than the control; AAI equal to 0% means the extract and control presented the same result in relation to the inhibition halos; AAI values in the range ]0 to 100%[ mean the extract showed higher inhibition halos than the control; and AAI equal to 100% means the control showed no inhibition halos.

This analysis was performed in triplicate with three independent assays.

### 2.6. Phytotoxicity Assay

For germination tests, each extract was first prepared with distilled water to a concentration of 0.30 g/mL and diluted to 0.03, 0.01, and 0.003 g/mL. From each extract concentration, 5 mL was placed in Petri dishes (Ø 10 cm) with filter paper, and 10 seeds of garden-cress (*Lepidium sativum* L., purchased from a local market) were placed equidistantly. Distilled water was used for the control under the same conditions. All Petri dishes were placed in an incubator at 25 °C for 5 days, protected from light. Every day the number of germinated seeds and the root length were recorded.

The results were analyzed according to the guidelines of EN 16086-1 and EN 16086-2 [39,40], where the Number of Germinated Seeds, Germination Rate (%), Root Length (mm), and the Munoo–Liisa Vitality Index (%) were determined. The Munoo–Liisa Vitality Index (Table 1) allowed for classifying the extracts according to their phytotoxicity.

### 2.7. Statistical Analysis

All data are expressed as mean values ± standard deviation. Statistical analysis was performed using GraphPad Prims Software version 8.0.2 (GraphPad Software, Inc., San Diego, CA, USA). The normality distribution of the data was evaluated by the Shapiro-Wilk test at a significance level of 5%. A three-way analysis of variance (3-way ANOVA) was applied to infer the statistical significance of the effects under study as well as the respective interactions in the bioactive and antimicrobial potentials results. If the 2-way and 3-way interaction effects were not significant, Tukey’s test was further used to determine the differences among means obtained for different samples. All analyses were performed at a 5% significance level. Correlations between parameters were established using Pearson’s correlation coefficient (*r*). A one-way analysis of variance (ANOVA) using Tukey’s test was performed to determine the differences between the means obtained in phytotoxicity analysis at significance level of 5%.

Principal component analysis (PCA) was performed using statistical program R (version 2.15.1), at a 5% significance level. This analysis was applied as an unsupervised pattern recognition tool to evaluate the overall potential of the EY, TPC, TF, TAN, ANT, AA, and inhibition halos data, determined based on conventional analytical techniques, to classify the extracts according to the pomegranate extraction method (conventional or sonication-assisted), by-product (peels or seeds), or the pomegranate cultivar (Acco, Big Full, and Wonderful).

## 3. Results and Discussion

### 3.1. Extraction Yield and Bioactive Compounds

The extraction yields (EY), total phenolic compounds (TPC), total flavonoids (TF), tannins (TAN), anthocyanins (ANT), and the antioxidant activity (AA, expressed as IC_50_) of the extracts of peels and seeds of Acco, Big Full, and Wonderful cultivars, obtained using the two studied extraction methods, are shown in Table 2. The three main effects under study (cultivar, by-product, and extraction method) significantly influenced the TPC, TF, ANT, and AA values (*p* < 0.0001). Since for each parameter under study one or more of the 2-way/3-way interactions were statistically significant (*p*-value < 0.05), the significance of the main effects could not be further interpreted based on the output of the post-hoc multicomparison tests. The EY and TAN were only affected by the cultivar and the by-product. The highest overall TPC was obtained with conventional extraction compared to the sonication-assisted extraction (0.16 to 0.73 mg GAE/mg extract vs. 0.11 to 0.50 mg GAE/mg extract, respectively). The TF, ANT, and AA were enhanced by the sonication-assisted extraction, reaching 0.019 to 0.068 mg CATE/mg extract for TF, 0.06 to 0.60 µg C3G/mg db for ANT, and 0.010 to 0.200 mg/mL for AA. Figure S1 presents a visual representation of the PCA results and the output clearly pointed out that each of the main effects considered could be effectively differentiated using an unsupervised PCA model based on the first three principal components (PCs).

A study conducted by Passafiume et al. [42] on pomegranate juice concluded that in terms of phenolic compounds and antioxidant activity, Wonderful cultivar presented higher values than the Acco cultivar, but the anthocyanin content of Acco juice was higher. These findings in the juice are similar to those found in this study for both peels and seeds extracts from the two mentioned cultivars. In terms of seeds, extracts from Big Full cultivar had the lowest bioactive levels, however, an opposite trend was observed for peels, as the Big Full extracts are those with the highest bioactive potential, independent of the extraction method used. Many studies point out that the Wonderful cultivar has the highest antioxidant activity amongst other cultivars [43]. In this study, the peels extract of the new cultivar Big Full surpasses not only the AA (in sonication-assisted extraction) of Wonderful’s extracts, but also the amounts of TPC, TF, TAN, and ANT (for both extractions methods).

It should be mentioned that the EY, TPC, and AA values found in the present study (Table 2) for peels extracted using the sonication-assisted extraction method are greater than those previously reported for pomegranate peels extracts also obtained by sonication by Tabaraki et al. [44], Sharayei et al. [45], and Ranjha et al. [46] (EY of 37.5%, TPC of 0.03–0.07 mg GAE/mg, and AA of 0.44 mg/mL IC_50_, by the DPPH method), but lower than those described by Bandara et al. [47] (EY of 38%, TPC of 0.64 mg GAE/mg, and AA of 0.003 mg/mL IC_50_). These findings clearly showed that different extraction conditions (frequency, time, solvent), as well as the pomegranate cultivar, greatly affects the extraction performance, which is useful to optimize the extraction conditions in each case under study. Overall, the results of Bandara et al. [47] suggest that higher frequency-time of extraction may enhance the amount of the extracted bioactive compounds.

It has been described that pomegranate fruit and its by-products have one of the highest antioxidant activities among other fruits and their by-products, being reported as a linear correlation between the phenolic content and the antioxidant activity [48,49,50,51,52,53]. Thus, for the bioactive compounds evaluated, the existence of linear correlations was assessed through the Pearson’s correlation test (Table 3). According to the Shapiro–Wilk test, with the exception of AA (*p*-value < 0.05), all variables presented a normal distribution (*p*-value ≥ 0.05) Since the antioxidant activity is inversely proportional to the IC_50_ of the extract, the correlations with AA are negative. As expected, strong correlations were found between TPC and TF (*r* = 0.723), TAN (*r* = 0.861), and AA (*r* = −0.789). The strongest AA correlation was observed with TAN and TF with coefficients (*r*) −0.893 and −0.862, respectively. These correlations have already been described, since the hydrolysable tannins and flavonoids of pomegranate peels can contribute to AA [54,55]. These findings are similar to those reported by Masci et al. [50], Yan et al. [56], and Orak et al. [55] in peels. ANT did not correlate with TPC, TF, and AA, but showed a negative correlation with TAN (*r* = −0.377). These results are in agreement with the studies of Orak et al. [55] on peels.

### 3.2. Antimicrobial Potential

Several studies have confirmed that pomegranate’s phenolics and flavonoids are related to a high antimicrobial potential against foodborne pathogens [48,57,58,59] and antifungal properties [22].

Table 4 shows the values of the inhibition halos obtained with the different extracts against the tested microorganisms (Figure S2 shows some examples of the inhibition halos obtained for the studied extracts). The inhibition halos obtained for the controls (penicillin, kanamycin, and amphotericin B) are given in Table S1. *E. coli* showed resistance to all extracts. Moreover, the use of Big Full seeds against *P. aeruginosa* and *B. cereus* was not effective. This may be attributed to the lowest bioactive potential of these extracts. Significant differences (*p* < 0.05) were found regarding the extraction method, where the sonication-assisted extraction originated the extracts with the highest inhibition halos against *P. aeruginosa* (5.6 to 13.0 mm), *S. aureus* (9.3 to 14.0 mm), and *Y. lipolytica* (9.2 to 13.8 mm). It was also observed that the type of by-product resulted in significantly different inhibition halos against *P. aeruginosa*, *S. aureus*, and *B. cereus* (*p* < 0.05).

Globally, the results found in this study regarding the pomegranate peel antimicrobial potential are in line with the literature data. McCarrell et al. [60] reported that pomegranate peel extracts (0.33 g/mL) did not inhibit *E. coli* or *P. aeruginosa* but inhibited *S. aureus* (14 mm), showing higher antimicrobial potential than some extracts obtained in the present study (7.9 to 14 mm). On the other hand, Panichayupakaranant et al. [61] showed that peels’ extracts (0.20 g/mL) did not inhibit *E. coli* but were effective against *S. aureus* (15.2 to 19.4 mm of halos), which is in agreement with the results presented in this study. Alexandre et al. [20] found that pomegranate peel extract (0.50 g/mL) inhibited *E. coli* (22 mm), *P. aeruginosa* (31 mm), *S. aureus* (22 mm), and *B. cereus* (19 mm), suggesting that the use of a higher extract concentrations can promote or enhance the antimicrobial capacity. The mentioned studies confirmed the findings of Silva et al. [62] and Hanani et al. [63], which described *S. aureus* as one of the most sensitive bacteria to pomegranate extracts, and this was also confirmed in the present work (7.9 to 11.5 mm for conventional extracts and 9.3 to 14.0 mm for sonication assisted extracts, Table 4).

With the results presented, it is also possible to expect that these extracts may possess bacteriostatic activity—it causes inhibition of bacterial growth but not death (external action required to cause death) [37,61].

Regarding antimicrobial activity against fungi, Rosa-Burgos et al. [64] and Hlima et al. [37] reported that pomegranate peels’ extracts inhibited the growth of several filamentous fungi (e.g., *Alternaria alternata*, *Aspergillus flavus*, *Aspergillus niger*, *Aspergillus parasiticus*, *Botrytis cinerea*, *Fusarium culmorum*, *Fusarium graminearum Fusarium oxysporum*, and *Fusarium verticillioides*), with inhibitions halos of 8 to 15 mm. Likewise, the present study showed that peel and seed extracts, obtained from both extraction methods and from any of the three cultivars, demonstrated antimicrobial activity against *Y. lipolytica* (8.3–15.7 mm), which is a single-cell fungus.

The fact that the extracts have demonstrated inhibition against the tested microorganisms (except *E. coli*) suggests that their compositions comprise a wide range of compounds with antimicrobial properties. Many mechanisms can lead to a higher antimicrobial activity, from the chemical composition of the matrix to the active compounds’ extraction method. For example, the sterilization of extracts by autoclaving appears to increase antimicrobial activity compared to sterilization by filtration [60].

The antimicrobial activity of pomegranate by-products may be indicative of the presence of metabolic toxins or a board spectrum of antibacterial compounds that act against Gram-positive and Gram-negative bacteria and yeasts [65]. In fact, Gullon et al. [66] suggest that the antimicrobial effects may be attributed to the combination of several bioactive compounds that cause microbial death through numerous mechanisms.

Thus, Pearson’s correlation was applied between the results of the bioactive compounds found in by-products’ extracts (TPC, TF, TAN, ANT, and AA) and the antimicrobial potential, based on the inhibition halos (Table 5). According to the Shapiro–Wilk test, with the exception of AA and the inhibition halos of *P. aeruginosa* (*p*-value < 0.05), all variables presented a normal distribution (*p*-value ≥ 0.05). Positive correlations were found for TPC, TF, and TAN with the inhibition halos for all microorganisms (*p* < 0.05), which showed that a higher bioactive potential led to a higher antimicrobial activity, although this straightforward finding was not always verified by other researchers. Furthermore, strong correlations between AA and the inhibition halos of *P. aeruginosa* (*r* = −0.902), *S. aureus* (*r* = −0.756) and *B. cereus* (*r* = −0.700) were found. As antimicrobial activity and AA are derived from the same compounds, it is expected there is a correlation between the two.

Table 6, Table 7 and Table 8 show the antimicrobial activity index (AAI) for the extracts in relation to kanamycin, penicillin, and amphotericin B, respectively. The AAI evaluates the inhibition halos caused by the extracts relatively to a control (antibiotic/antifungal). The inhibition halos caused by kanamycin, penicillin, and amphotericin B are presented in Table S1.

Regarding AAI for kanamycin (Table 6), all results are negative indicating that this antibiotic had a higher inhibition (greater inhibition halos) than the tested extracts. *S. aureus* presented the values closer to 0%, since the diameter of the inhibition halos of the extracts are closer to the halos originated by kanamycin, while *E. coli* gave values of −100% because, contrary to kanamycin, none of the extracts inhibited this Gram-negative bacterium. This means that kanamycin has a greater potential of inhibition against the studied microorganisms than any of the extracts tested.

Regarding AAI for penicillin (Table 7), the values ranged from −100% to 100%. Once again, *E. coli* presented an AAI of −100% because no inhibition halos were detected for the extracts. For *P. aeruginosa*, all values were negative because penicillin had a greater inhibitory effect than the evaluated extracts. Some extracts, most of which peel extracts, promoted higher inhibition halos compared to penicillin, showing a greater inhibition against *S. aureus* (positive values for AAI). Since penicillin presented no effect on *B. cereus*, the extracts reached an AAI of 100% (with the exception of the Big Full seed extract).

The highest values of AAI are reported for the extracts relatively to amphotericin B (Table 8). The positive values revealed that all extracts caused more inhibition of *Y. lipolytica* than the antifungal used as the positive control.

### 3.3. Phytotoxicity Assay

Despite the beneficial characteristics of pomegranate by-products, it is important to remark that like any other plant extract, extracts from pomegranate peel or seeds can also present toxicity [59]. The evaluation of the toxicity of the extracts is of paramount importance when applied to foods, which was evaluated by studying the phytotoxicity of the extracts obtained. Thus, in case of phytotoxicity, these results may also allow a glimpse of their possible application as natural herbicides in agriculture. To the authors’ best knowledge, no studies have been conducted to evaluate the phytotoxicity of pomegranate peel extracts. For this assay, the germination test was conducted with garden-cress seeds submitted to different concentrations of Acco, Big Full, and Wonderful peel extracts, from the two studied extractions methods (conventional and sonication-assisted). Only the peels’ extracts were selected for this test, since they had greater bioactive and antimicrobial potential than the seeds. Garden-cress seeds (*Lepidium sativum*) were chosen based on the work of Luo et al. [67], where it is described that these seeds are the most used for phytotoxicity studies.

To understand which concentration of the extract did not cause toxicity to the garden-cress seeds, a preliminary test was performed with different concentrations of the extracts: 0.30 g/mL (100%), 0.03 g/mL (10%), 0.01 g/mL (3.33%), and 0.003 g/mL (1%). The highest concentration was chosen based on the antimicrobial assays, since it was the concentration that inhibited different pathogenic microorganisms. For five days, the number of germinated seeds and the root length were monitored and compared to the control, for which distilled water was used (Figure S3).

It was found that the concentration of 0.30 g/mL totally inhibited seed germination; the concentration of 0.03 g/mL partially inhibited germination; and only the lowest concentrations (0.01 and 0.003 g/mL) allowed the seeds’ germination. Regarding the root length, at 0.30 and 0.03 g/mL, there was no root growth for any of the seeds. At the concentration of 0.01 g/mL, only part of the roots managed to grow. Only at the lowest extract concentration (0.003 g/mL) were acceptable roots for all seeds developed. Thus, the concentration of 0.003 g/mL (1% of the initial one) was chosen for further tests.

The germination rate of 100% was observed for all peel extracts with a concentration of 0.003 g/mL, regardless of the pomegranate cultivar or extraction method applied. Only the Wonderful peel extract obtained by conventional extraction had a lower germination rate (96.7%). Regarding root length (Table 9), the extracts obtained by the sonication-assisted extraction method were the ones that least affected root growth. Even so, the control showed roots with twice of the length of the roots submitted to the extracts (*p*-value < 0.0001), which indicated that they inhibited the growth of garden-cress roots. For both extraction methods, extracts from cultivar Acco showed the lowest inhibition of root growth.

The Munoo-Liisa vitality index at the fifth day of the root growth is presented in Table 10. According to the phytotoxicity classification of Trautmann and Krasny [41] (Table 1), all extracts at the concentration 0.003 g/mL can be classified as phytotoxic for garden-cress seeds, except the extracts from Big Full and Wonderful cultivars, obtained by conventional extraction, which are considered very phytotoxic.

The observed phytotoxicity can be attributed to the phenolic compounds present in the extracts, which are phytotoxic and may also be responsible for plant necrosis if present in higher concentrations [68,69]. Furthermore, the phytotoxicity effect of phenolic compounds seemed to be related to their lipophilic or hydrophilic character, as it has been reported that lipophilic phenolics can cause greater phytotoxicity than hydrophilic ones [70]. A Pearson’s correlation was performed between the bioactive compounds and the root length of the garden-cress seeds on the fifth day of exposure to the peel extracts, but no significant correlations were found (*p* > 0.05). However, the correlation of root length with TPC was negative, indicating that higher amounts of phenolics can inhibit root length development, as previously reported.

## 4. Conclusions

In general, for both peels and seeds, the sonication-assisted extraction method, using EtOH 50% as a solvent, proved to be more effective for the extraction of the bioactive compounds (TF and ANT) of pomegranate by-products, leading to extracts with higher AA and antimicrobial potential and lower phytotoxicity. In relation to TPC, conventional extraction was more effective, and TAN extraction is not influenced by the extraction method. Pomegranate peels showed promising bioactive and antimicrobial activities compared to seeds. Among the cultivars studied, the new cultivar Big Full showed the best bioactive potential. In terms of antimicrobial activity, extracts at a concentration of 0.30 g/mL inhibit *P. aeruginosa*, *S. aureus*, *B. cereus*, and *Y. lipolytica*, but not *E. coli*. Regarding phytotoxicity studies, all peel extracts were highly phytotoxic, even at very low concentrations (0.003 g/mL). Since it is described that antioxidant activity, antimicrobial activity, and phytotoxicity can be attributed to the phenolic compounds, it would be important to determine the phenolic profile of each extract in order to understand the cause–effect of these compounds in relation to the biological activities of the extracts.

This study contributed to increase the knowledge about the antimicrobial properties of pomegranate seed extracts and the phytotoxicity of the peel extracts, since studies are scarce in the literature. In addition, data regarding the Big Full cultivar are reported for the first time, showing the promising potential of its by-products. Moreover, the results regarding the phytotoxicity of pomegranate peels can contribute to expanding their possible applications in the agricultural sector, as possible natural substitutes for herbicides.

## Figures and Tables

**Table 1:** Phytotoxicity classification (adapted from [41]).

Munoo–Liisa Vitality Index (%)	Classification
>100	Enhances germination and root growth
80–100	Nonphytotoxic
60–80	Moderately phytotoxic
40–60	Phytotoxic
<40	Very phytotoxic

**Table 2:** Extraction yield (EY, %, db), total phenolic compounds (TPC, mg GAE/mg extract), total flavonoids (TF, mg CATE/mg extract), tannins (TAN, % *w*/*w*, db), anthocyanins (ANT, µg C3G/mg, db), and antioxidant activity (AA expressed in terms of IC_50_, mg/mL, db) of the peels and seeds of three pomegranate cultivars (Acco, Big Full, Wonderful) according to extraction method (conventional vs. sonication-assisted).

Extraction Method	By-Product	Cultivar	EY	TPC	TF	TAN	ANT	AA (IC_50_)
Conventional	Peels	Acco	49.9 ± 0.9	0.39 ± 0.02	0.029 ± 0.002	16.7 ± 0.6	0.05 ± 0.02	0.024 ± 0.000
		Big Full	51.0 ± 0.3	0.73 ± 0.18	0.052 ± 0.003	25.3 ± 0.2	0.12 ± 0.01	0.180 ± 0.005
		Wonderful	46.3 ± 4.8	0.32 ± 0.01	0.042 ± 0.005	18.7 ± 0.3	0.00 ± 0.01	0.022 ± 0.001
	Seeds	Acco	59.2 ± 1.3	0.21 ± 0.03	0.008 ± 0.000	3.5 ± 0.5	0.14 ± 0.01	0.063 ± 0.004
		Big Full	35.7 ± 1.2	0.16 ± 0.03	0.007 ± 0.001	1.8 ± 0.3	0.22 ± 0.02	0.398 ± 0.017
		Wonderful	32.0 ± 1.7	0.23 ± 0.00	0.021 ± 0.001	9.6 ± 1.0	0.08 ± 0.01	0.042 ± 0.003
Sonication	Peels	Acco	49.1 ± 0.4	0.37 ± 0.04	0.038 ± 0.004	16.1 ± 1.0	0.18 ± 0.03	0.024 ± 0.013
		Big Full	54.9 ± 1.2	0.50 ± 0.05	0.047 ± 0.002	26.7 ± 1.4	0.29 ± 0.03	0.010 ± 0.000
		Wonderful	47.8 ± 0.5	0.33 ± 0.00	0.038 ± 0.002	18.3 ± 0.5	0.06 ± 0.05	0.021 ± 0.001
	Seeds	Acco	48.9 ± 1.3	0.14 ± 0.00	0.019 ± 0.002	3.5 ± 0.7	0.28 ± 0.03	0.067 ± 0.003
		Big Full	36.8 ± 0.4	0.11 ± 0.00	0.032 ± 0.004	1.3 ± 0.3	0.60 ± 0.13	0.200 ± 0.001
		Wonderful	30.8 ± 1.0	0.19 ± 0.02	0.068 ± 0.010	8.5 ± 0.2	0.18 ± 0.08	0.030 ± 0.000
*p*-value	Cultivar (A)		<0.0001	<0.0001	<0.0001	<0.0001	<0.0001	<0.0001
	By-product (B)		<0.0001	<0.0001	<0.0001	<0.0001	<0.0001	<0.0001
	Extraction method (C)		0.0778	0.0005	<0.0001	0.3244	<0.0001	<0.0001
	A × B interaction		<0.0001	<0.0001	<0.0001	<0.0001	0.0038	<0.0001
	A × C interaction		<0.0001	0.0083	0.0009	0.0869	<0.0001	<0.0001
	B × C interaction		<0.0001	0.4289	<0.0001	0.1395	0.0045	0.0652
	A × B × C interaction		0.0244	0.0139	<0.0001	0.0829	0.0086	0.0777

Results are expressed as mean values ± standard deviation of four independent extractions for EY (*n* = 4) and duplicates of two independent extractions for TPC, TF, TAN, ANT, and IC_50_ (*n* = 4). Three-way ANOVA (*p*-value < 0.05).

**Table 3:** Pearson’s correlation coefficient (*r*) and the related significance between the total phenolic compounds (TPC), total flavonoids (TF), tannins (TAN), anthocyanins (ANT), and antioxidant activity (AA, IC_50_).

	TPC	TF	TAN	ANT	AA
TPC	1.000				
TF	0.723 **	1.000			
TAN	0.861 **	0.854 **	1.000		
ANT	−0.320	−0.073	−0.377 *	1.000	
AA	−0.789 *	−0.862 **	−0.893 **	0.209	1.000

* *p*-value < 0.05, ** *p*-value < 0.0001.

**Table 4:** Inhibition halos (mm) for each extract vs. microorganism tested.

Extraction Method	By-Product	Cultivar	*E. coli*	*P. aeruginosa*	*S. aureus*	*B. cereus*	*Y. lipolytica*
Conventional	Peels	Acco	R	12.3 ± 1.0	10.6 ± 0.7	10.8 ± 0.7	11.5 ± 1.0
		Big Full	R	10.4 ± 1.9	11.5 ± 1.5	13.4 ± 1.7	15.7 ± 0.8
		Wonderful	R	10.5 ± 0.5	11.3 ± 0.6	11.1 ± 1.2	9.2 ± 0.9
	Seeds	Acco	R	2.9 ± 4.4	9.7 ± 0.9	7.6 ± 0.7	9.2 ± 0.9
		Big Full	R	R	7.9 ± 0.6	R	8.3 ± 1.5
		Wonderful	R	9.7 ± 0.7	11.2 ± 1.4	10.2 ± 0.8	10.1 ± 1.6
Sonication	Peels	Acco	R	13.0 ± 1.2	11.3 ± 1.4	11.7 ± 1.8	13.8 ± 1.5
		Big Full	R	11.6 ± 2.0	14.0 ± 0.9	15.5 ± 1.5	11.4 ± 2.2
		Wonderful	R	11.4 ± 1.9	13.1 ± 0.6	11.2 ± 0.7	10.7 ± 1.2
	Seeds	Acco	R	5.6 ± 4.6	10.2 ± 1.5	8.4 ± 0.8	10.7 ± 2.0
		Big Full	R	R	9.3 ± 0.7	R	9.2 ± 1.3
		Wonderful	R	10.3 ± 1.2	10.7 ± 0.9	6.6 ± 5.1	12.2 ± 1.8
*p*-value	Cultivar (A)		-	<0.0001	0.0009	<0.0001	0.0826
	By-product (B)		-	<0.0001	<0.0001	<0.0001	<0.0001
	Extraction method (C)		-	0.0111	0.0002	0.9024	0.0202
	A × B interaction		-	<0.0001	<0.0001	<0.0001	<0.0001
	A × C interaction		-	0.6016	0.1894	0.0020	<0.0001
	B × C interaction		-	0.5583	0.0025	0.0066	0.0049
	A × B × C interaction		-	0.3813	0.2031	0.1033	0.0002

R: resistant. Results are expressed as mean values ± standard deviation of triplicates of three independent assays (*n* = 9). Three-way ANOVA (*p*-value < 0.05).

**Table 5:** Pearson’s correlation coefficient (*r*) and significance between the bioactive potential of each extract and the inhibition halos caused by pomegranate extracts against the tested microorganisms.

	*P. aeruginosa*	*S. aureus*	*B. cereus*	*Y. lipolytica*
TPC	0.621 *	0.605 *	0.750 *	0.764 *
TF	0.672 *	0.740 *	0.647 *	0.664 *
TAN	0.811 *	0.840 **	0.868 **	0.624 *
ANT	−0.607 *	−0.301	−0.557	−0.178
AA	−0.902 **	−0.756 *	−0.700 *	−0.403

TPC, total phenolic compounds; TF, total flavonoids; TAN tannins; ANT, anthocyanins; AA, antioxidant activity (IC_50_). * *p*-value < 0.05, ** *p*-value < 0.001.

**Table 6:** Antimicrobial activity index (%) for peels and seeds extracts of Acco, Big Full, and Wonderful cultivars, from both extractions, relative to kanamycin.

Extraction Method	By-Product	Cultivar	*E. coli*	*P. aeruginosa*	*S. aureus*	*B. cereus*
Conventional	Peels	Acco	−100.0 ± 0.0	−29.7 ± 2.7	−16.3 ± 0.4	−21.9 ± 3.1
		Big Full	−100.0 ± 0.0	−37.1 ± 4.8	−12.5 ± 4.4	−11.5 ± 5.4
		Wonderful	−100.0 ± 0.0	−36.8 ± 1.2	−13.1 ± 0.7	−20.6 ± 5.6
	Seeds	Acco	−100.0 ± 0.0	−81.3 ± 32.3	−20.6 ± 1.9	−38.0 ± 2.9
		Big Full	−100.0 ± 0.0	−100.0 ± 0.0	−30.3 ± 2.0	−100.0 ± 0.0
		Wonderful	−100.0 ± 0.0	−40.3 ± 1.2	−13.7 ± 4.4	−24.4 ± 3.2
Sonication	Peels	Acco	−100.0 ± 0.0	−28.5 ± 2.5	−16.2 ± 5.8	−23.8 ± 8.6
		Big Full	−100.0 ± 0.0	−34.1 ± 8.9	−5.6 ± 1.2	−9.7 ± 2.3
		Wonderful	−100.0 ± 0.0	−34.3 ± 4.5	−9.1 ± 0.4	−25.3 ± 2.2
	Seeds	Acco	−100.0 ± 0.0	−63.4 ± 31.9	−21.6 ± 7.1	−38.1 ± 3.6
		Big Full	−100.0 ± 0.0	−100.0 ± 0.0	−25.7 ± 2.6	−100.0 ± 0.0
		Wonderful	−100.0 ± 0.0	−38.4 ± 3.3	−18.8 ± 3.6	−54.4 ± 40.0

Results are expressed as mean values ± standard deviation of three replicates (*n* = 3).

**Table 7:** Antimicrobial activity index (%) for peels and seeds extracts of Acco, Big Full, and Wonderful cultivars, from both extractions, relative to penicillin.

Extraction Method	By-Product	Cultivar	*E. coli*	*P. aeruginosa*	*S. aureus*	*B. cereus*
Conventional	Peels	Acco	−100.0 ± 0.0	−33.4 ± 2.6	8.7 ± 0.5	100.0 ± 0.0
		Big Full	−100.0 ± 0.0	−40.7 ± 4.7	12.5 ± 4.4	100.0 ± 0.0
		Wonderful	−100.0 ± 0.0	−40.3 ± 1.2	11.9 ± 0.7	100.0 ± 0.0
	Seeds	Acco	−100.0 ± 0.0	−82.4 ± 30.4	4.3 ± 2.0	100.0 ± 0.0
		Big Full	−100.0 ± 0.0	−100.0 ± 0.0	−6.2 ± 2.2	0.0 ± 0.0
		Wonderful	−100.0 ± 0.0	−43.7 ± 1.2	11.3 ± 4.5	100.0 ± 0.0
Sonication	Peels	Acco	−100.0 ± 0.0	−34.3 ± 2.4	−6.4 ± 5.9	100.0 ± 0.0
		Big Full	−100.0 ± 0.0	−39.7 ± 8.4	4.3 ± 1.2	100.0 ± 0.0
		Wonderful	−100.0 ± 0.0	−39.9 ± 4.3	0.9 ± 0.3	100.0 ± 0.0
	Seeds	Acco	−100.0 ± 0.0	−66.5 ± 29.0	−11.9 ± 7.3	100.0 ± 0.0
		Big Full	−100.0 ± 0.0	−100.0 ± 0.0	−16.1 ± 2.7	0.0 ± 0.0
		Wonderful	−100.0 ± 0.0	−43.8 ± 3.1	−9.0 ± 3.7	100.0 ± 0.0

Results are expressed as mean values ± standard deviation of three replicates (*n* = 3).

**Table 8:** Antimicrobial activity index (%) for peels and seeds extracts of Acco, Big Full, and Wonderful cultivars, from both extractions, relatively to amphotericin B.

Extraction Method	By-Product	Cultivar	*Y. lipolytica*
Conventional	Peels	Acco	67.9 ± 1.0
		Big Full	75.4 ± 0.5
		Wonderful	61.4 ± 3.2
	Seeds	Acco	61.2 ± 2.8
		Big Full	56.8 ± 6.3
		Wonderful	64.0 ± 4.4
Sonication	Peels	Acco	42.1 ± 1.5
		Big Full	33.4 ± 9.1
		Wonderful	31.3 ± 0.4
	Seeds	Acco	31.0 ± 6.0
		Big Full	23.0 ± 5.7
		Wonderful	36.7 ± 4.4

Results are expressed as mean values ± standard deviation of three replicates (*n* = 3).

**Table 9:** Root length (mm) of garden-cress seeds, in the presence of water (control) and pomegranate peel extracts (0.003 g/mL).

Extraction Method	Cultivar	Day 1	Day 2	Day 3	Day 4	Day 5
Control		<5.0	15.0 ± 2.0 a	27.2 ± 3.2 a	36.5 ± 3.4 a	41.0 ± 3.2 a
Conventional	Acco	<5.0	7.5 ± 1.3 b	11.4 ± 2.6 bc	14.6 ± 4.3 bc	17.4 ± 5.7 bc
	Big Full	<5.0	6.5 ± 1.5 b	9.5 ± 0.5 b	12.5 ± 2.3 c	14.6 ± 0.7 b
	Wonderful	<5.0	7.1 ± 0.7 b	11.9 ± 1.7 bc	14.0 ± 1.2 c	15.7 ± 1.4 b
Sonication	Acco	<5.0	7.7 ± 1.1 b	14.3 ± 3.6 c	19.8 ± 5.9 c	23.4 ± 4.8 c
	Big Full	<5.0	7.0 ± 1.1 b	14.3 ± 1.2 c	18.0 ± 2.5 bc	20.7 ± 3.4 bc
	Wonderful	<5.0	7.6 ± 0.4 b	15.2 ± 1.3 c	17.6 ± 1.3 bc	19.0 ± 1.6 bc
*p*-value		-	<0.0001	<0.0001	<0.0001	<0.0001

Results are expressed as mean values ± standard deviation of 10 replicates of three independent assays (*n* = 30). One-way ANOVA (*p*-value < 0.0001). Different small letters in the same column represent statistical differences between extracts and the control on the same day.

**Table 10:** Munoo-Liisa vitality index (%) for pomegranate peel extracts (0.003 g/mL) and phytotoxicity classification.

Extraction Method	Cultivar	Day 5	Phytotoxicity Classification
*Conventional*	Acco	42.4 ± 13.9	Phytotoxic
	Big Full	35.7 ± 1.7	Very phytotoxic
	Wonderful	37.1 ± 5.4	Very phytotoxic
*Sonication*	Acco	57.1 ± 11.8	Phytotoxic
	Big Full	50.5 ± 8.2	Phytotoxic
	Wonderful	46.3 ± 3.9	Phytotoxic

Results are expressed as mean values ± standard deviation of three replicates (*n* = 3).

## Data Availability

Data are contained within this article.

## Supplementary Materials

The following are available online at https://www.mdpi.com/article/10.3390/foods11070992/s1, Table S1: Inhibition halos (mm) caused by penicillin, kanamycin (antibiotics), and amphotericin B (antifungal) on the tested microorganisms (*n* = 18). Figure S1: 3D-PCA plots for the pomegranate extracts classification based on the experimental analysis according to: (A) the extraction method (conventional or sonication-assisted); (B) the type of by-product (peels or seeds); and (C) the cultivar (Acco, Big Full, or Wonderful). Figure S2: Inhibition halos formed due to the antimicrobial activity of the studied extracts: (A) schematic representation of a Petri dish with extracts (E), water (W), kanamycin (K), and penicillin (P); (B) inhibition halos of Acco peel extracts (sonication extraction) against *P. aeruginosa*; (C) inhibition halos of Big Full peel extracts (conventional extraction) against *S. aureus*; and (D) inhibition halos for Wonderful peel extracts (sonication extraction) against *B. cereus*. Figure S3. Preliminary phytotoxicity test against garden-cress seeds with extracts from Wonderful peel (sonication extraction) to determine (A) the number of germinated seeds and (B) the root length (mm) of the germinated seeds.
