# Supplementary material for: Effect of Extraction Method on the Bioactive Composition, Antimicrobial Activity and Phytotoxicity of Pomegranate By-Products
Source: Foods. 2022 Mar 29;11(7):992. doi: 10.3390/foods11070992 (PMC8997943; doi:10.3390/foods11070992)
Supplement: Supplementary file 1 [file foods-11-00992-s001.zip › foods-1585183-supplementary.pdf]

Supplementary Material

Table S1. Inhibition halos (mm) caused by penicillin, kanamycin (antibiotics) and amphotericin B (anti-fungal) on the tested microorganisms (n=18).

	Conventional Extraction			Sonication-assisted Extraction		
	**Penicillin**	**Kanamycin**	**Amphotericin B**	**Penicillin**	**Kanamycin**	**Amphotericin B**
E. coli	2.2 ± 2.7	15.6 ± 5.9	-	4.3 ± 2.5	17.5 ± 0.8	-
P. aeruginosa	24.7 ± 2.5	22.7 ± 2.5	-	26.6 ± 3.2	23.3 ± 0.9	-
S. aureus	8.9 ± 2.3	14.8 ± 1.3	-	12.8 ± 1.7	15.7 ± 0.7	-
B. cereus	R	16.8 ± 0.9	-	R	18.8 ± 2.4	-
Y. lipolytica	-	-	2.2 ± 1.9	-	-	5.6 ± 1.9

R: resistant.

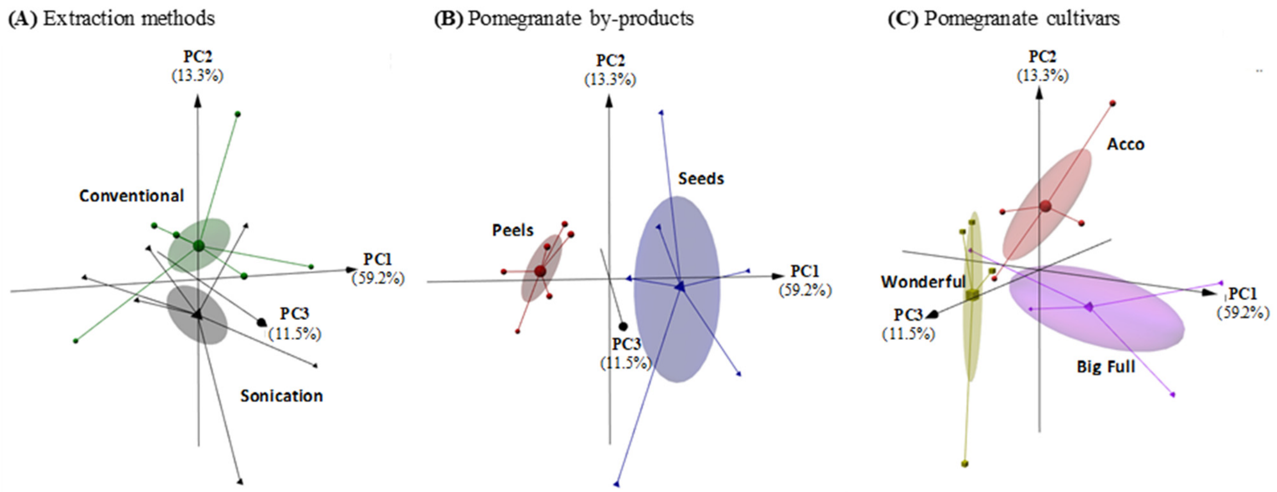

Figure S1. 3D-PCA plots for the pomegranate extracts classification based on the experimental analysis according to: (A) the extraction method (conventional or sonication-assisted); (B) the type of by-product (peels or seeds); and (C) the cultivar (Acco, Big Full or Wonderful).

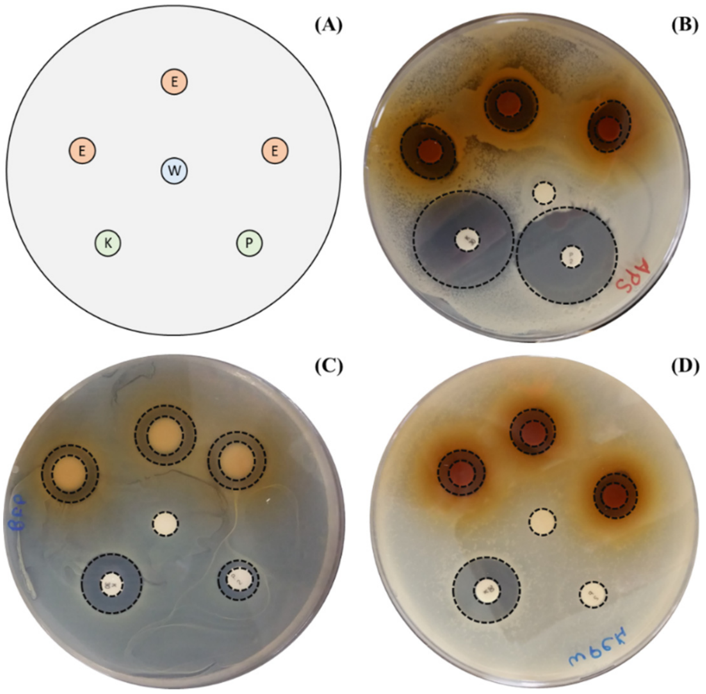

Figure S2. Inhibition halos formed due to the antimicrobial activity of the studied extracts: **(A)** schematic representation of a Petri dish with extracts (E), water (W), kanamycin (K) and penicillin (P); **(B)** inhibition halos of Acco peel extracts (sonication extraction) against *P. aeruginosa*; **(C)** inhibition halos of Big Full peel extracts (conventional extraction) against *S. aureus*; and, **(D)** inhibition halos for Wonderful peel extracts (sonication extraction) against *B. cereus*.

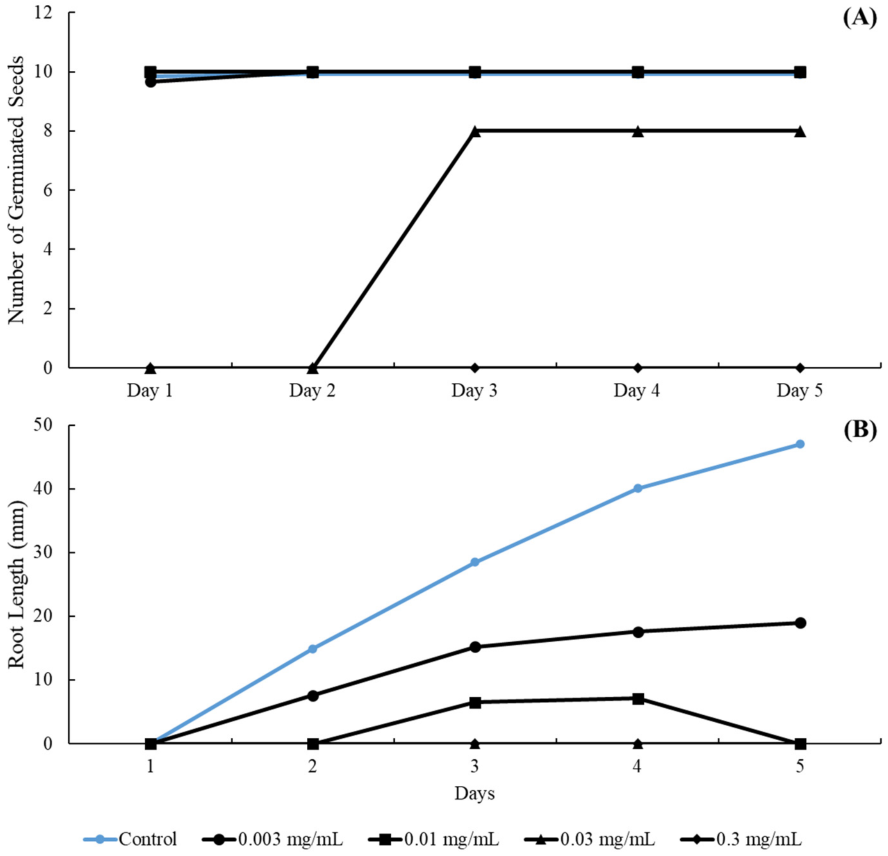

Figure S3. Preliminary phytotoxicity test against garden-cress seeds with extracts from Wonderful peel (sonication extraction) to determine **(A)** the number of germinated seeds; and **(B)** roots length (mm) of the germinated seeds.
